# Myc suppression of *Nfkb2 *accelerates lymphomagenesis

**DOI:** 10.1186/1471-2407-10-348

**Published:** 2010-07-02

**Authors:** Ulrich Keller, Jürgen Huber, Jonas A Nilsson, Mohammad Fallahi, Mark A Hall, Christian Peschel, John L Cleveland

**Affiliations:** 1 III. Medical Department, Technische Universität München, Munich, Germany; 2 Department of Biochemistry, St. Jude Children's Research Hospital, Memphis, Tennessee, USA; 3 Department of Anesthesiology, Technische Universität München, Munich, Germany; 4 Department of Molecular Biology, Umeå University, Umeå, Sweden; 5 Department of Cancer Biology, The Scripps Research Institute, Scripps Florida, Jupiter, Florida, USA

## Abstract

**Background:**

Deregulated c-Myc expression is a hallmark of several human cancers where it promotes proliferation and an aggressive tumour phenotype. Myc overexpression is associated with reduced activity of Rel/NF-κB, transcription factors that control the immune response, cell survival, and transformation, and that are frequently altered in cancer. The Rel/NF-κB family member *NFKB2 *is altered by chromosomal translocations or deletions in lymphoid malignancies and deletion of the *C*-terminal ankyrin domain of NF-κB2 augments lymphocyte proliferation.

**Methods:**

Precancerous Eμ-*Myc*-transgenic B cells, Eμ-*Myc *lymphomas and human Burkitt lymphoma samples were assessed for *Nfkb2 *expression. The contribution of *Nfkb2 *to Myc-driven apoptosis, proliferation, and lymphomagenesis was tested genetically in vivo.

**Results:**

Here we report that the Myc oncoprotein suppresses *Nfkb2 *expression in vitro in primary mouse fibroblasts and B cells, and in vivo in the Eμ-*Myc *transgenic mouse model of human Burkitt lymphoma (BL). *NFKB2 *suppression by Myc was also confirmed in primary human BL. Promoter-reporter assays indicate that Myc-mediated suppression of *Nfkb2 *occurs at the level of transcription. The contribution of *Nfkb2 *to Myc-driven lymphomagenesis was tested in vivo, where *Nfkb2 *loss was shown to accelerate lymphoma development in Eμ-*Myc *transgenic mice, by impairing Myc's apoptotic response.

**Conclusions:**

*Nfkb2 *is suppressed by c-Myc and harnesses Myc-driven lymphomagenesis. These data thus link Myc-driven lymphomagenesis to the non-canonical NF-κB pathway.

## Background

Members of the Rel/NF-κB family of transcription factors, including RelA (p65), RelB, c-Rel, NF-κB1 [p105/p50] and NF-κB2 [p100/p52], form homodimers and heterodimers that control aspects of T and B cell development, proliferation and survival. The activity of NF-κB dimers is held in check by dedicated inhibitors coined IκBα or IκBβ, which bind to and sequester NF-κB in the cytoplasm. Signals that activate NF-κB promote phosphorylation and ubiquitin-mediated destruction of the IκBs, which are substrates of the upstream IκB kinases Ikkα or Ikkβ (reviewed in [1-3]).

Regulation of NF-κB2 is unique amongst NF-κB family members. First, p100 NF-κB2 protein harbors a *C*-terminal ankyrin repeat domain that has intrinsic IκB activity, whereas its Rel homology domain directs its nuclear translocation, dimerization, and DNA binding functions. Second, p100 is proteolytically processed to p52, an event that requires Nik (NF-κB-inducing kinase) and Ikkα [4,5] activity. Finally, targeted deletion of *Nfkb2 *in mice leads to defects in B cell development and in lymphoid organogenesis [6].

NF-κB promotes cell survival and proliferation, and alterations in this pathway, via chromosomal translocation or amplification, mutations, and deletions are common in cancer [7-9]. Important roles for *NFKB2 *in lymphomagenesis have been implicated from studies in both mouse and man. First, *NFKB2 *is a common target of chromosomal rearrangements in human B lymphomas, and these typically truncate the protein, generating constitutively active nuclear forms [10,11] that have increased transcriptional activity compared to p52 [12,13]. Furthermore, loss of the *C*-terminal ankyrin domain of NF-κB2 in mice results in enlarged lymph nodes and augments lymphocyte proliferation [14].

The *c-MYC *gene (hereafter Myc) is the cellular homolog of v-Myc, the transforming gene of the MC29 avian leukemia virus [15]. Myc oncoproteins (c-Myc, N-Myc and L-Myc) are activated in ~70% of human malignancies [16] and they function as basic helix-loop-helix-leucine zipper transcription factors that coordinate cell growth, division and metabolism [17-19]. In normal tissue, the regulation of Myc transcription and turnover is tightly controlled by mitogenic and growth inhibitory cues [20], and in tumour cells these controls are frequently lost, either by chromosomal translocations or amplifications, or indirectly by mutations in regulatory pathways.

The Rel/NF-κB pathway is suppressed in Myc-driven human Burkitt lymphoma (BL) [21] and in Myc overexpressing precancerous B cells [22]. Here we report that Myc suppresses *Nfkb2 *expression in B cells and show that NF-κB2 contributes to Myc's apoptotic response that harnesses lymphomagenesis.

## Methods

### Mice and tumour analyses

*Nfkb2 *null mice (C57BL/6) [6] (obtained from Christopher Hunter, University of Pennsylvania, Philadelphia, PA, USA) were bred with Eμ-*Myc *transgenic mice (C57Bl/6) [23]. F_1 _Eμ-*Myc*;*Nfkb2*^+/- ^offspring were bred to *Nfkb2*^*+/- *^mice to obtain the desired Eμ-*Myc;Nfkb2*^*+/+ *^(n = 80) and Eμ-*Myc*;*Nfkb2*^*-/- *^(n = 44) cohort. Note that numbers of Eμ-*Myc*;*Nfkb2*^*-/- *^mice generated were lower than those of Eμ-*Myc;Nfkb2*^*+/+ *^mice due to integration of the *Myc *transgene on chromosome 19 where the *Nfkb2 *gene resides (assessed by FISH analysis, data not shown). Animals were observed for signs of morbidity and tumour development. Lymphomas were harvested, snap-frozen in liquid nitrogen, and then processed for RNA and protein analyses.

With Institutional Review Board approval, and following informed consent, lymphomas from 17 BL patients were banked. RNA and protein were extracted from these tumours (kindly provided by Drs. John Sandlund and Mihaela Onciu, St. Jude Children's Research Hospital, SJCRH, Memphis, TN). As a control, pooled peripheral blood mononuclear cells (PBMC) from healthy donors were enriched using CD19-MicroBeads (Miltenyi Biotech, Bergisch-Gladbach, Germany) and RNA and protein were prepared.

### Cell culture

Primary bone marrow (BM)-derived pre-B cells were cultured as described [24]. B cells and mouse embryonic fibroblasts (MEFs, cultured from E13.5-E14.5 embryos) were infected with MSCV-Myc-ER™-IRES-GFP or MSCV-Myc-IRES-GFP retroviruses as described [25]. To activate Myc, Myc-ER™-expressing cells were treated with 2-μM 4-hydroxy-tamoxifen (4-HT) and lysed for protein and RNA analyses. P493-6 human B cells were kindly provided by G. Bornkamm (Helmholtz Zentrum München, Munich, Germany) and were cultured with tetracycline (Tet) or estrogen (both from Sigma-Aldrich, Taufkirchen, Germany) as described [26]. To suppress *Myc *transcription, cells were treated with Tet (Sigma, Taufkirchen, Germany) for 60 hr; to reactivate *Myc *cells were then resuspended in medium lacking Tet. Transient transfections of HeLa cells were performed using Lipofectamine 2000 according to the manufacturer's instructions (Invitrogen, Carlsbad, CA).

### FACS analysis and magnetic-activated cell sorting (MACS) of B cells

Rates of proliferation of bone marrow (BM) and splenic B220^+^sIgM^+ ^and B220^+^sIgM^- ^cells were determined using a Flow Kit as described by the manufacturer (BD Biosciences Pharmingen, San Diego, CA). Briefly, animals were injected intraperitoneally with 100 μl of 10 mg/ml BrdU in sterile PBS. Animals were humanely sacrificed 12 hr following injection and BM and spleen were harvested. Red cells were lysed using ammonium chloride/potassium bicarbonate solution. Cells were then resuspended, incubated with antibody against B220 and sIgM (BD Biosciences Pharmingen, San Diego, CA), washed and collected by centrifugation. One million cells were further processed and stained with anti-BrdU-FITC antibody, and 5 × 10^5 ^cells were stained with Propidium iodide and Annexin-V FITC antibody (Annexin-V-Fluor Kit, Roche Applied Sciences, Indianapolis, IN). Following incubation cells were washed, resuspended in PBS, and then analysed by FACS. The remainder of the BM and spleen cells were enriched for B cells by magnetic cell sorting with B220 MicroBeads according to the manufacturer's instructions (Miltenyi Biotech, Bergisch-Gladbach, Germany) and were lysed for immunoblot or real-time PCR analysis.

### RNA preparation and analyses

RNA was prepared using the RNeasy kit (Qiagen, Valencia, CA). For Affymetrix analyses, cRNA was synthesized using the One-Cycle Target Labeling and Control Reagent package (Affymetrix Inc., Santa Clara, CA) and the reaction was probed to the 430A mouse Affymetrix chip. The GCRMA normalization algorithm was applied to all Affymetrix Chips using GeneSpring GX (v7.3). GeneSpring Hierarchical Clustering (Similarity measure: Pearson Correlation, Clustering Algorithm: Average Linkage) was applied to those probe sets with signal higher than median in at least one sample. For real-time PCR, cDNA was prepared from 1 μg RNA using the iScript cDNA synthesis kit (Bio-Rad, Hercules, CA). Real-time PCR was performed using an iCycler machine (Bio-Rad) and the iTaq SYBR green kit (Bio-Rad). Data analysis was performed by comparing Ct values with a control sample set as 1. Sequences of primers are available upon request.

### Immunoblotting

Protein extracts (20 or 50 μg per lane) were electrophoretically separated on SDS-PAGE gels, transferred to membranes (Protran, Schleicher & Schuell, Dassel, Germany) and blotted with antibodies specific for NF-κB2, c-Myc, IKKα, Miz-1 (Santa Cruz Inc., Santa Cruz, CA) and β-Actin (Sigma Chemicals, St. Louis, MO). Equal loading of protein was demonstrated by Ponceau Red staining of the nitrocellulose membranes following transfer.

### Reporter assay

HeLa cells were co-transfected with an *Nfkb2 *promoter-reporter construct (*firefly *luciferase, gift of R. M. Schmid, Munich, Germany) and *p65 *(R. M. Schmid), c-*Myc *or *GFP *expression plasmids. Relative luciferase activity was determined as described by the manufacturer (Promega, Madison, WI), by calculating the ratio of *firefly *to co-transfected *renilla *luciferase activity.

## Results

### The Rel/NF-κB pathway is suppressed in Myc-induced lymphomas

**Figure 1:**
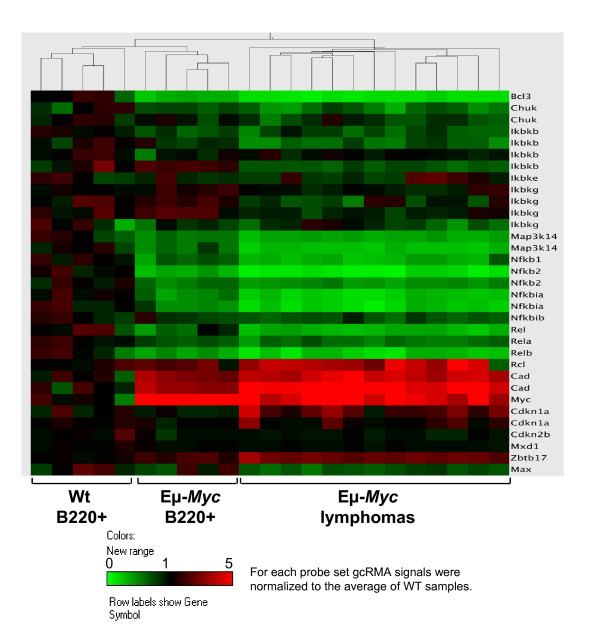
**Myc targets the Rel/NF-κB network in precancerous B cells and lymphomas**. Hierarchical clustering of *Rel/Nfkb *family members, upstream kinases that regulate the NF-κB pathway, and selected control genes was performed using RNA prepared from B220^+ ^splenic B cells from five weanling-age wild type mice (wt), from five Eμ-*Myc *transgenic mice (Eμ-*Myc*), and from thirteen Eμ-*Myc *lymphomas. Probe set signals were normalized to the mean across wt samples, and values of each individual sample are represented by a colour, with green corresponding to expression below, red corresponding to expression above, and black corresponding to expression equal to the wt mean expression.

In the Eμ-*Myc *model of human B cell lymphoma disease progression is characterized by a pre-neoplastic state in which high proliferative rates of Eμ-*Myc *B cells are offset by high levels of apoptosis, which is then disabled during progression to the malignant state [24,27]. The Rel/NF-κB pathway is suppressed in *Myc*-transgenic precancerous B cells [22] and in human B lymphoma [21], yet the underlying targets in this response are not resolved. To address this issue we initially assessed the expression of NF-κB components in B220^+ ^splenic B cells of 4 week-old precancerous Eμ-*Myc *transgenic mice (*n *= 5) and their wild type littermate controls (*n *= 5), and in several (*n *= 13) Eμ-*Myc *lymphomas. As expected, there were clear increases in the levels of the established Myc targets *Cad *[28] and *Rcl *[29], in precancerous Eμ-*Myc *B cells, and even more so in Eμ-*Myc *lymphomas, compared to their levels in wild type splenic B cells (Figure 1, lower panel). In contrast, there were reduced levels of nearly all mRNAs encoding *Rel/Nfkb *factors, their inhibitors (*Nfkbia *and *Nfkbib*), and their upstream regulators in pre-malignant Eμ-*Myc *B cells compared to their levels expressed in wild type B cells (Figure 1). Strikingly, this response was significantly augmented in frank Eμ-*Myc *lymphomas (Figure 1). Thus, Myc-mediated suppression of the Rel/NF-κB pathway is augmented following the switch to the neoplastic state.

### *Nfkb2 *RNA and protein levels are suppressed by c-Myc

**Figure 2:**
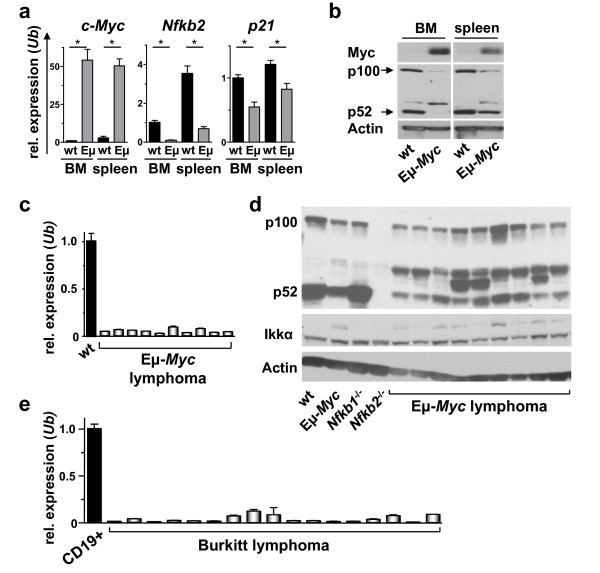
***Nfkb2 *suppression in precancerous Myc-transgenic B cells and Myc-driven lymphomas**. a) real-time PCR analysis of *c-Myc*, *Nfkb2 *and *p21 *mRNA levels in bone marrow (BM) and splenic (spleen) B220^+ ^wild type (wt, grey bars) and precancerous Eμ-*Myc *(Eμ, black bars) B cells. Levels of mRNA are standardized to the expression of *Ubiquitin *(*Ub*), which is not regulated by Myc. * indicates p < 0.05. b) immunoblot analyses of the indicated proteins in control and precancerous Eμ-*Myc *B220^+ ^B cells from bone marrow (BM) and spleen. c) real-time PCR analysis of *Nfkb2 *expression in ten Eμ-Myc lymphomas or splenic wild type (wt) B220^+ ^B cells. d) immunoblot analysis of NF-κB2 p100 and p52 expression in wild type (wt) and Eμ-*Myc *precancerous B220^+ ^B cells and Eμ-*Myc *lymphomas. *Nfkb2*^-/- ^and *Nfkb1*^-/- ^B220^+ ^B cells served as controls. e) real-time PCR analysis of *NFKB2 *expression in seventeen human Burkitt lymphoma samples compared to CD19^+ ^control B cells. Levels of mRNAs were normalized to the expression of *Ubiquitin *(*Ub*).

*NFKB2 *is the most frequently mutated *Rel*/*Nfkb *member in human lymphoid malignancies [10,13]. We thus assessed the effects of Myc on *Nfkb2 *expression in B cells by real time PCR and western blot analyses of age-matched littermate B220^+ ^wild type and precancerous Eμ-*Myc *B cells. Consistent with the profiling data, *Nfkb2 *mRNA and NF-κB p100 and p52 protein levels were markedly reduced in Eμ-*Myc *bone marrow (BM) and splenic B220^+ ^B cells compared to wild type B cells (Figure 2a, b). *Nfkb2 *suppression was not due major differences in B cell subsets in the BM or spleens of wild type versus pre-malignant Eμ-*Myc *mice (data not shown). Further, there was a marked suppression of *Nfkb2 *transcripts in all Eμ-*Myc *lymphomas (Figure 2c), and in all human Burkitt lymphomas compared to pooled CD19^+ ^control B cells from healthy donors (Figure 2e). As expected, Myc transcript and protein levels remained highly elevated in manifest lymphoma [22,30]. Finally, both NF-κB p100 and p52 levels were reduced in the majority of Eμ-*Myc *lymphomas (Figure 2d). Thus, *Nfkb2 *expression is suppressed in *Myc*-driven lymphomas in mice and man.

### *Nfkb2 *transcription is repressed by Myc

The regulation of *Nfkb2 *gene expression is complex and involves positive and negative regulatory mechanisms, including autoregulation [31,32]. In part its expression is controlled by the activity of Ikkα [33]. However, we did not observe significant changes of Ikkα protein in Eμ-*Myc *lymphomas (Figure 2d). Thus, other mechanisms must mediate the suppression of *Nfkb2 *by Myc in B cells.

**Figure 3:**
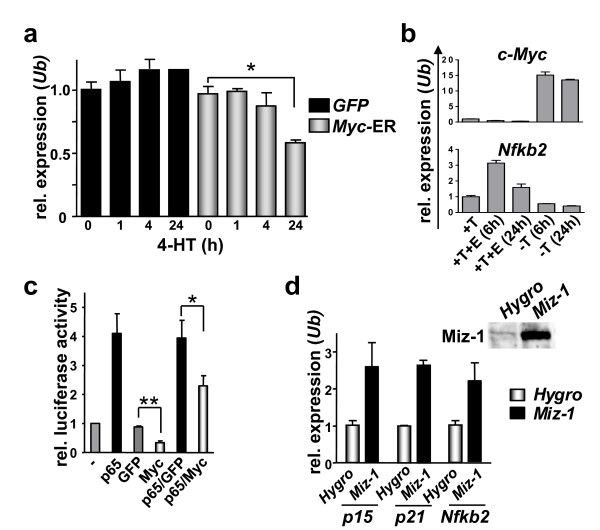
**Myc represses *Nfkb2 *transcription**. a) SYBR-green real-time PCR analysis of *Nfkb2 *mRNA levels in primary, ex vivo cultured B cells infected with MSCV-*Myc*-ER™-IRES-*GFP *(*Myc*-ER) virus or MSCV-IRES-*GFP *(*GFP*) control virus. GFP-positive cells were sorted by flow cytometry, cultured, and treated with 4-hydroxytamoxifen (4-HT) for the indicated times. Levels of mRNAs are standardized to the expression of *Ubiquitin *(*Ub*). * indicates p < 0.05. b) SYBR-green real-time PCR analysis of *NFKB2 *expression in human P493-6 cells treated with tetracycline (T, Myc-off state) ± estrogen (E, EBNA2-on state) for the indicated times. c) HeLa cells were co-transfected with an *Nfkb2 *promoter-reporter construct (*firefly *luciferase) and the indicated expression plasmids. The relative luciferase activity was determined by calculatiing the ratio of *firefly *to co-transfected *renilla *luciferase activity. * indicates p < 0.05, ** indicates p < 0.01. d) expression of the indicated transcripts measured by real-time PCR following infection of primary MEFs with *Miz-1 *or control (*Hygro*) retroviruses. Levels of the indicated mRNA were standardized to the expression of *Ubiquitin *(*Ub*).

To confirm that *Nfkb2 *expression in B cells was indeed responsive to Myc, primary BM-derived B cells from wild type mice were cultured and infected with the MSCV-Myc-ER™-IRES-GFP retrovirus that expresses the tamoxifen-regulated, estrogen receptor chimeric Myc transgene, Myc-ER™ [34], along with the gene encoding green fluorescence protein (GFP) through the agency of an internal ribosome entry site (IRES). GFP + cells were isolated by FACS and expanded in culture in IL-7 medium. These cells were then treated with 4-hydroxytamoxifen (4-HT) to activate Myc-ER™. Notably, Myc activation led to reductions in the levels of *Nfkb2 *transcripts (Figure 3a). To exclude possible indirect effects of Myc-induced apoptosis on *Nfkb2 *expression, we also evaluated human P493-6 B cells, which harbor a tetracycline (Tet) promoter-regulated, conditional human c-*Myc *transgene [26] that is robustly induced following removal of Tet. Notably the induction of c-Myc in this system led to marked reduction in *Nfkb2 *mRNA levels (Figure 3b). Finally, using the Myc-ER™ system, we also assessed *Nfkb2 *mRNA in primary mouse embryonic fibroblasts (MEFs). Again, *Nfkb2 *mRNA levels were reduced following activation of Myc-ER™ (Additional File 1). Therefore, the activation or overexpression of Myc suppresses *Nfkb2 *mRNA and protein expression.

To test whether *Nfkb2 *transcription was repressed by Myc, we assessed the effects of Myc on *Nfkb2 *promoter activity using luciferase reporter-based plasmids [32] in HeLa cells. Notably, Myc significantly repressed *Nfkb2*-promoter activity, and co-expression of Myc also inhibited the robust induction of the *Nfkb2 *promoter by p65/RelA (Figure 3c). Myc often represses transcription by binding to and inhibiting the functions of the transcriptional activator Miz-1 [35]. Since the *Nfkb2 *promoter region contains an Initiator element (INR)-like sequence and INRs mediate Miz-1-induced transcription [36], we evaluated whether Miz-1 overexpression induced *Nfkb2 *transcripts. As expected, the direct Miz-1 target genes *p15*^*INK4b *^and *p21 *[37,38] were induced by Miz-1 in primary MEFs, and *Nfkb2 *transcript levels were also increased (Figure 3d). Thus, the Myc-mediated suppression of *Nfkb2 *transcription may involve disruption of Miz-1 functions.

### *Nfkb2 *impairs Myc-induced lymphomagenesis

**Figure 4:**
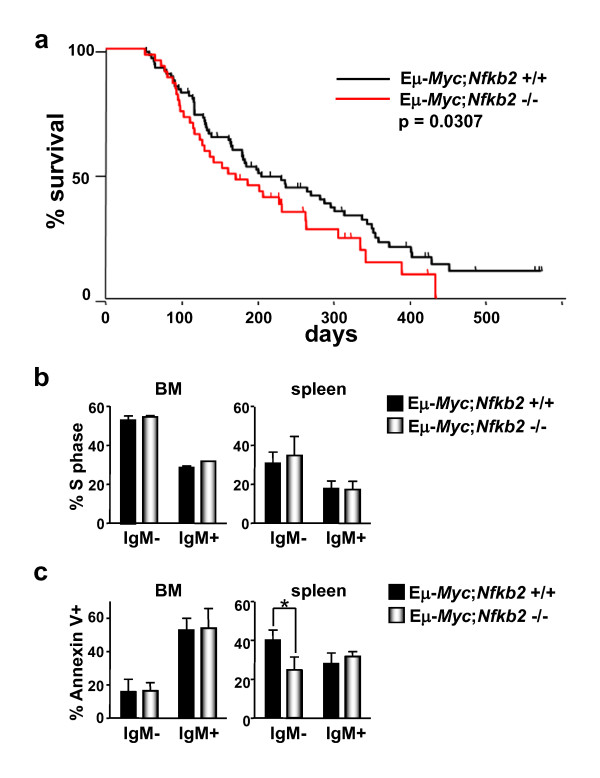
***Nfkb2 *loss accelerates Myc-induced lymphomagenesis by impairing apoptosis**. a) Effects of *Nfkb2 *deficiency on Myc-induced lymphomagenesis. The survival of Eμ-*Myc *transgenic littermates of the indicated *Nfkb2 *genotypes is shown. The differences in the rates of tumor incidence between the *Nfkb2*^*+/+ *^and the *Nfkb2*^*-/- *^littermates is statistically significant (p = 0.0307). b) Eμ-*Myc *transgenic littermates of the indicated *Nfkb2 *genotypes were injected with BrdU, and cells from bone marrow and spleen were harvested 12 hr later. BrdU-incorporation was analyzed using an antibody-dependent fluorescence assay. The bars show the mean percentage of cells in S phase ± SEM (three independent experiments). c) The apoptotic index of the indicated genotypes was analyzed using an antibody dependent fluorescence assay. Annexin V^+ ^B220^+ ^cells of sIgM^- ^or sIgM^+ ^phenotype are shown. The bars represent the mean ± SEM from three independent experiments. * indicates p < 0.05.

The remarkable changes in the expression of components of the Rel/NF-κB signalling pathway, and particularly the suppression of *Nfkb2 *by c-Myc, suggested that NF-kB2 might play critical roles in Myc-driven tumorigenesis. If Myc-mediated reductions in *Nfkb2 *expression in Eμ-*Myc *B cells were important for Myc-mediated lymphomagenesis, we reasoned that total loss of *Nfkb2 *should affect lymphoma development. To test this hypothesis, Eμ-*Myc *transgenic mice were mated to *Nfkb2*^-/- ^mice [6] and F1 offspring were bred to *Nfkb2*^+/- ^mice to obtain the desired Eμ-*Myc*;*Nfkb2*^+/+ ^and Eμ-*Myc*;*Nfkb2*^-/- ^cohorts. These littermates were then observed for lymphoma onset. Eμ-*Myc *transgenic mice usually succumb to aggressive lymphoma within 4-8 months of birth [23]. As expected, non-transgenic *Nfkb2*^-/- ^littermates showed no signs of tumour development throughout their lifespan (data not shown). Importantly, Eμ-*Myc*;*Nfkb2*^-/- ^transgenic displayed a moderately accelerated course of lymphoma development and, accordingly, had a shorter lifespan, with a median survival of 171 days compared to 205 days median survival in their Eμ-*Myc*;*Nfkb2*^+/+ ^littermates (Figure 4a, p = 0.0307). The lymphomas that arose in Eμ-*Myc*;*Nfkb2*^-/- ^transgenics were phenotypically identical to those that arose in Eμ-*Myc*;*Nfkb2*^+/+ ^mice, and full necropsy and histopathological examination demonstrated similar dissemination of disease in Eμ-*Myc*;*Nfkb2*^+/+ ^versus Eμ-*Myc*;*Nfkb2*^-/- ^mice (data not shown). Thus, *Nfkb2 *loss accelerates Myc-driven lymphomagenesis without overtly affecting the disease phenotype.

Loss of the tumour suppressors p53 or Arf, which mediate Myc's apoptotic response [25], dramatically accelerates Myc-driven tumorigenesis [24,39]. By contrast, loss of regulators of the Myc-to-p27^Kip1 ^pathway that regulates Myc's proliferative response, such as E2f1 and Cks1, markedly delays lymphoma development and prevents dissemination of disease [27,30,40]. We therefore assessed the effects of *Nfkb2 *loss on Myc's proliferative and apoptotic responses. To evaluate effects on Myc's proliferative response BrdU was injected intraperitoneally into 4-week old Eμ-*Myc*;*Nfkb2*^+/+ ^and Eμ-*Myc*;*Nfkb2*^-/- ^littermates and after 12 hr B220^+^sIgM^+ ^and B220^+^sIgM^- ^cells were assessed for their S phase indices. Loss of *Nfkb2 *had essentially no effects on Eμ-*Myc *B cell proliferation in either BM or spleen (Figure 4b). To address the effects of *Nfkb2 *loss on Myc-induced apoptosis, the apoptotic indices of pre-cancerous B220^+ ^B cells from Eμ-*Myc*;*Nfkb2*^+/+ ^and Eμ-*Myc*;*Nfkb2*^-/- ^mice were determined by staining with anti-Annexin V-FITC and propidium iodide. There was a significant reduction in the apoptotic indices of sIgM^- ^splenic Eμ-*Myc*;*Nfkb2*^-/- ^B cells compared to matched sIgM^- ^splenic B cells from Eμ-*Myc*;*Nfkb2*^+/+ ^mice (Figure 4c). The sIgM^- ^population of B cells are those most prone to Myc-induced apoptosis in the Eμ-*Myc *model [41]. Thus, the accelerated lymphoma development manifest in Eμ-*Myc*;*Nfkb2*^-/- ^mice is associated with an impaired apoptotic response.

## Discussion

Members of the Rel/NF-κB family can, depending on cell context, act as either tumour suppressors or oncogenes [42]. A hallmark of human Burkitt lymphoma, and of pre-malignant B cells and lymphomas of Eμ-*Myc *mice, is suppression of the Rel/NF-κB signaling pathway [21,22,43]. These findings, and those reported herein, suggest that Myc-induced suppression of this important immune and inflammatory regulatory network contributes to lymphomagenesis.

Myc executes most of its functions as a transcription factor that regulates a large cast of target genes (see: Myc Cancer Gene http://www.myc-cancer-gene.org; reviewed in [44,45]). Genes suppressed by Myc include those encoding proteins involved in apoptosis [19] and growth arrest [46]. Various mechanisms of transcriptional repression by Myc are operational. Amongst these, Myc-mediated inhibition of Miz-1 transcriptional activity by Myc:Max heterodimers is clearly a central pathway by which Myc overrides cell growth control [44-46]. Growth arrest genes, including *p21 *[47], *p15 *[37] and *Gadd45 *[48], are elevated in c-*Myc*^-/- ^cells and are suppressed in cells that overexpress Myc. In B lymphocytes Myc is required for normal B cell proliferation and CD40-mediated proliferation [49]. CD40 signalling has been shown to activate the non-canonical NF-κB pathway [50]. It was therefore tempting to speculate that the suppression of *Nfkb2 *by Myc controls the expansion of B cells under normal and/or stressful conditions. Unexpectedly, in the Eμ-*Myc *model precancerous B cell proliferation was not significantly reduced upon *Nfkb2 *loss.

The recently described autoimmune disease in mice having constitutive NF-κB2 p52 in lymphocytes is associated with increased B cell proliferation and a defective response to apoptotic stimuli [51]. Further, in transgenic mice that express the lymphoma-associated NF-κB2 mutant p80HT, B cell proliferation is not affected but lymphomas do occur, and B cells from these mice are resistant to apoptosis [52]. In the Eμ-*Myc *model loss of *Nfkb2 *does not affect Myc's proliferative response, but it does disable the apoptotic response in sIgM^- ^Eμ-*Myc *B cells, which are the subset that is most prone to Myc-induced apoptosis in this mouse model [41]. Thus, at least in the context of Myc overexpression, which is clearly a hallmark of most rapidly dividing human malignancies including lymphoma and leukemia, NF-κB2 contributes to the apoptosis response and, accordingly, *Nfkb2 *loss accelerates Myc-driven lymphomagenesis. In addition to Myc suppression of the non-canonical pathway discovered herein, others have recently attributed tumour suppressive functions to classical NF-κB activity in the context of Myc-induced lymphoma [53]. Therefore, both the canonical and the non-canonical NF-κB pathway function as tumour suppressors in Myc-transformed murine lymphoma and most likely human Burkitt lymphoma.

## Conclusions

Our work identifies *Nfkb2 *as a Myc repression target, and Myc appears to regulate both basal and stimulated *Nfkb2 *transcription. Suppression of the non-canonical NF-κB pathway provides a selective advantage to Myc-transformed lymphomas and thus contributes to lymphomagenesis. These findings have implications for the development of therapies against Myc-dependent tumours.

## Competing interests

The authors declare that they have no competing interests.

## Authors' contributions

UK, CP and JLC designed the experiments. UK, JH and JAN carried out experiments as well as the statistical data analysis. MAH and MF performed gene expression analyses. UK and JLC wrote the manuscript. All authors read and approved the final manuscript.

## Pre-publication history

The pre-publication history for this paper can be accessed here:

http://www.biomedcentral.com/1471-2407/10/348/prepub

## Supplementary Material

Additional file 1 **Myc suppresses *Nfkb2 *expression in primary, early passage MEFs**. SYBR-green real-time PCR analysis of *Nfkb2 *RNA levels in primary MEFs infected with MSCV-*Myc*-ER™-IRES-*GFP *(*Myc*-ER) retrovirus. GFP-positive cells were sorted by flow cytometry and treated with 2 μM 4-HT for the indicated times. The known Myc targets *E2f1 *[27] and *p27 *[54] were included as controls. The levels of the mRNAs were standardized to the expression of *Ubiquitin *(*Ub*).* indicates p < 0.05. Click here for file
