# Supplementary figures and images for: Myc suppression of Nfkb2 accelerates lymphomagenesis
Source: BMC Cancer. 2010 Jul 2;10:348. doi: 10.1186/1471-2407-10-348 (PMC2902445; doi:10.1186/1471-2407-10-348)

## Slide 1
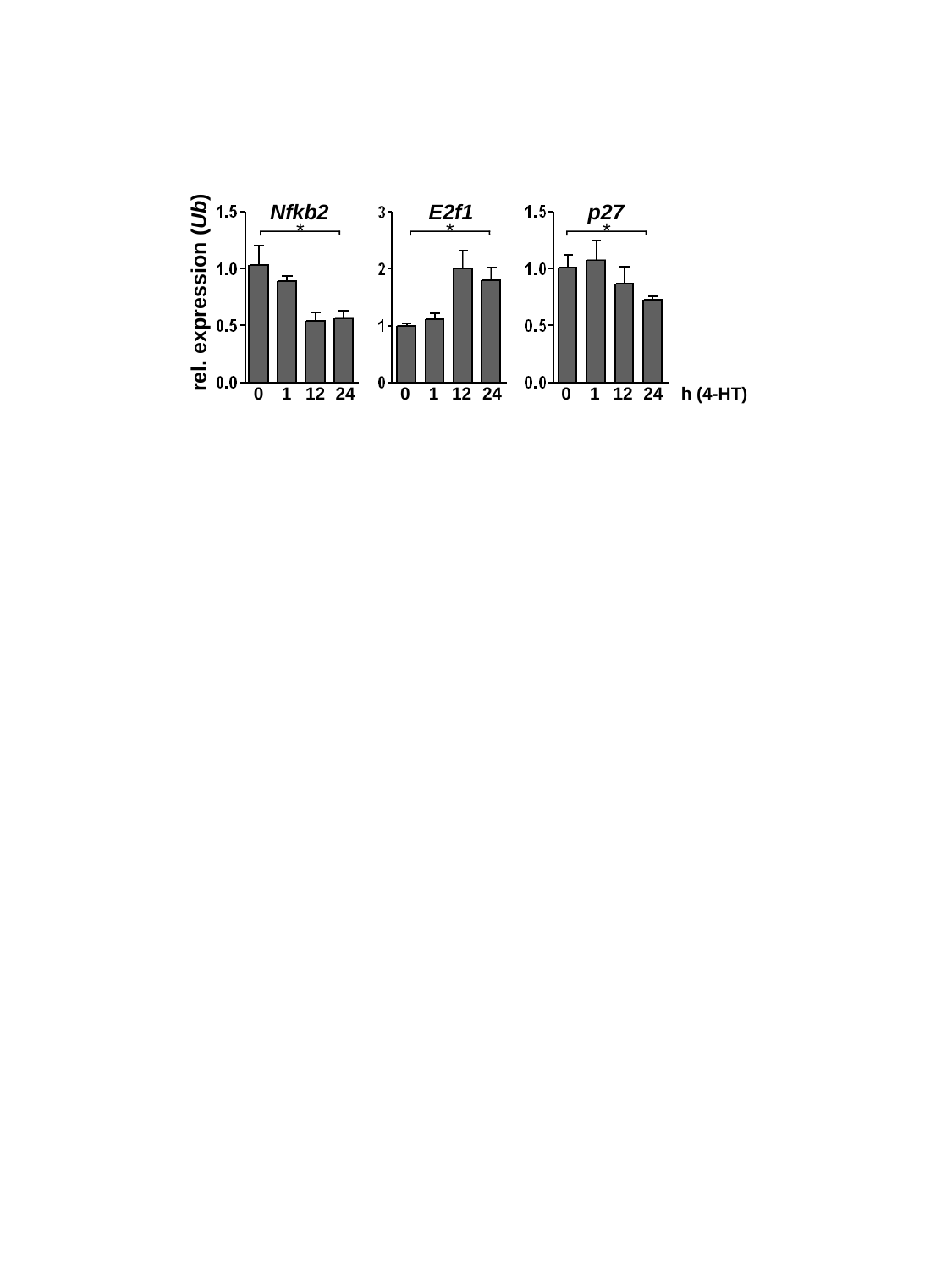

Nfkb2
E2f1
p27
*
*
*
rel. expression (Ub)
0
12
0
12
0
12
h (4-HT)
1
24
1
24
1
24

Supplement: Additional file 1 — Myc suppresses Nfkb2 expression in primary, early passage MEFs. SYBR-green real-time PCR analysis of Nfkb2 RNA levels in primary MEFs infected with MSCV-Myc-ER™-IRES-GFP (Myc-ER) retrovirus. GFP-positive cells were sorted by flow cytometry and treated with 2 μM 4-HT for the indicated times. The known Myc targets E2f1 [27] and p27 [54] were included as controls. The levels of the mRNAs were standardized to the expression of Ubiquitin (Ub).* indicates p < 0.05. [file 1471-2407-10-348-S1.PPT]
